# The reported colour formation mechanism in pitaya fruit through co-accumulation of anthocyanins and betalains is inconsistent and fails to establish the co-accumulation

**DOI:** 10.1186/s12864-022-08957-z

**Published:** 2022-11-09

**Authors:** Mohammad Imtiyaj Khan

**Affiliations:** Biochemistry and Molecular Biology Lab, Department of Biotechnology, Gauhati University, Assam 781014 Guwahati, India

**Keywords:** Anthocyanins, Betalains, Gene expression, Amaranthin, Gomphrenin-I

## Background

The premise of the paper authored by Zhou et al. [1] published in BMC Genomics is that, in pitayas *Hylocereus undatus* (red peel-red pulp or RR; green peel-white pulp or GW) and *H. megalanthus* (yellow peel-white pulp or YW, also called *Selenicereus megalanthus*, http://legacy.tropicos.org/Name/50251405?tab=acceptednames, accessed on 14/11/2020) [2, 3]), anthocyanins and betalains co-accumulate, and hence both contribute to peel and pulp colour formation. Transcriptome sequencing, metabolome analysis, and qPCR were carried out. Despite inconsistencies, incomplete data, and inaccurate interpretation of data in the paper, the authors concluded that anthocyanins and betalains might co-accumulate in the same plant. Recently, a similar claim of co-accumulation of anthocyanins and betalains in *Hylocereus* spp. was systematically refuted [4]. In nature, anthocyanins and betalains have been found to be mutually exclusive [5, 6]. However, it is possible that some plants may accumulate both the pigments. Herein, I systematically point out the inconsistencies and misinterpretations of data in the paper by Zhou et al. [1], to demonstrate that this study does not disprove the mutual exclusiveness of anthocyanins and betalains.

## Main text

### Profiling betacyanins and amaranthin do not meet established standards

White pulp of *H. undatus* fruits have been reported to contain no betacyanins and betaxanthins [7–10]. Since betalains were detected in white pulp of *H. undatus* by Zhou et al. [1], the analysis they carried out should be confirmed in accordance with the available standard practices [11]. Nevertheless, they did not report any confirmatory data, except referring to two previous studies among which only one reported data on white and red species of *Hylocereus* [9], which was contrary to the findings of Zhou et al. [1]. This indicates that the analytical data presented by Zhou et al. [1] was unreliable. What furthers this assumption is that no *Hylocereus sp*. has been reported to accumulate amaranthin and/or contain more gomphrenin-I than betanin and hylocerenin [12–15], unlike what Zhou et al. reported [1]. In fact, in none of the references that Zhou et al. [1] depended upon for secondary metabolite identification by declaring “…….metabolites with similar fragment ions were suggested to be the same compounds. (page 15, column 2, lines 5–6)”, there was detection of either amaranthin or gomphrenin-I in pitaya samples. However, Zhou et al. [1] reported the contrary without thorough chemical/spectral characterisation. Such characterisation steps include profiling authentic reference compounds, fragmentation or neutral loss patterns, and matching of tentative structures’ precursor *m*/*z* to relevant databases. Accurate mass determination based on isotopic abundance and various charged and adduct ion forms must be performed to rule out multiple candidate structures of a single molecular formula [11, 16]. Moreover, multiple analytical steps/techniques for confirmation would be absolutely required, because the only difference in the structures of gomphrenin-I and betanin is the position of glucose moiety attachment, and hence MS spectra of these two compounds are the same. Therefore, there is need for additional spectroscopic characterisation to confirm the identity of gomphrenin-I. However, quite questionably, on page 11, column 2, it is reasoned with regard to the presence of high gomphrenin-I that “……this is possibly due to the conversion of betanin into gomphrenin-I as the latter was the significantly enriched metabolite mapped on the betalain biosynthesis pathway.” This explanation has no scientific basis because the literature cited to support the explanation is a review paper focussed on betalain evolution in which there is no mention of gomphrenin-I and the (bio)chemistry of its conversion into betanin. With regard to betanin content, on page 11, column 2, the authors mention that “…[betanin] was present in low quantities in RR-peel as compared to GW and YW-peels…the quantity [of betanin] in GW pulp was almost double than RR pulp.” This cannot be reconciled with the betalain biosynthetic pathway, and also not supported by the metabolite profile provided in Table S9 in [1], i.e. RR pulp has more than 800 times total betacyanins than GW pulp, and RR peel has more than 5 times than GW peel. As mentioned above, *H. undatus* white pulp has been reported to contain no betaxanthins or betacyanins, let alone betanin. Therefore, it is contradictory that green samples and yellow peels had more betanin than red samples that had the highest betacyanin content among all studied samples. Contents of both betacyanins (ca. 1.5 mg/100 g fresh peels) and betaxanthins (ca. 7 mg/100 g fresh peels) in *H. megalanthus* have already been reported from China [17], whereas Colombian *Selenicereus megalanthus* (or *H. megalanthus*) peels were reported to contain ca. 2.5 mg betaxanthins/100 g fresh weight [18]. The quantification of betacyanins, in particular, and metabolites, in general, in Zhou et al. [1] considers the area under the peak, whose relationship with concentration could be established only through a linear regression curve of the respective authentic reference compound. The cascading effect of the absence of authentic reference compounds, and lack of proper identification of metabolites through spectral characteristics, could be seen in the case of metabolite profile of different samples, viz. pulp and peel of RR, GW and YW. For example, green peel and white pulp samples of *H. undatus* are expected to have the least betacyanin content, as Israeli *H. undatus* (white pulp) has been reported to contain no betacyanins and betaxanthins [10]. Surprisingly, Zhou et al. [1] reported green peel and white pulp to have total betacyanins content higher than yellow pulp and yellow peel, when the areas under the curve of all the major identified betacyanins in Table S9 [1] are summed up. As for YW pulp, Ecuadorian *S. megalanthus* (or *H. megalanthus*) pulp was earlier reported to contain no betacyanins and betaxanthins [10]. In addition, there are 433 metabolites listed in Table S9 [1], but, curiously, some of the commonly reported amino acids or amines, like L-DOPA, dopamine, and also ascorbic acid are not among them, though their presence in *H. megalanthus* [17, 19] and *H. undatus* [7–9, 19] has been established beyond doubt.

### Reported betalain biosynthetic gene expression and betalain- especially amaranthin- accumulation cannot be reconciled

The expression patterns of unigenes of betalain biosynthetic pathway presented in Fig. 6 [1] do not corroborate with betacyanin content presented in Table S9 [1]. Of the four genes, viz. CYP76AD1-like (Cluster-864.132907), *Portulaca grandiflora* DOD (Cluster-864.102567), *Beta vulgaris* DOD (Cluster-864.111172) and *Bougainvillea spectabilis* cD5GT (Cluster-864.24834), in RR peel and pulp, only the expression of CYP76AD1-like seems to correlate with their metabolite contents. That is, RR pulp has higher betacyanin content and CYP76AD1-like expression than RR peel. All the remaining genes were either less expressed or not significantly different in RR pulp than RR peel. Similarly, RR pulp had comparable or lower betalain biosynthetic gene expression than GW pulp, but total betacyanins content was much higher in RR pulp. Therefore, of all the four reported betalain biosynthetic genes, only the expression of CYP76AD1-like can be reconciled with the metabolite profile. However, based on Fig. 3 [1], CYP76AD1-like expression should lead to L-DOPA formation, but L-DOPA was not detected in any of the studied samples (Table S9 [1]). Further, based on Fig. 3 in [1], CYP76AD1-like gene was not expressed in GW pulp and peel, and hence betalain biosynthesis should not occur therein. Contrastingly, Table S9 [1] shows that GW samples have higher betalain content than corresponding YW samples. In addition, all the 21 genes whose expressions are listed in Fig. 3 [1] have lower or similar expressions in YW pulp and GW pulp compared to YW peel and GW peel. So, contrary to what Fig. 3 [1] suggests, i.e. betalain formation does not occur in the absence of CYP76ADs, thereby resulting in white pulp and green peel, all the above-mentioned observations do not support GW samples having higher betacyanin content than YW samples.

In Fig. 6 [1], none of the betalain biosynthetic genes presented has higher expression in RR peel or pulp as compared to GW pulp or peel. In fact, cluster-864.102567 (PgDOD-like) and cluster-864.111172 (uncharacterised protein or BvDOD-like) are less expressed in both RR peel and RR pulp, while the other genes remained not significantly different from that of corresponding GW samples. Therefore, the gene expression pattern does not support the metabolite profile, and it cannot be explained by focussing only on betanin content in the samples, as done on page 11, column 2, lines 8–13.

Gomphrenin-I is synthesised by a 6-*O*-GT in plants, particularly betacyanin-accumulating ones (as reviewed in [5]). Therefore, if there were no 6-*O*-GTs expressed and only 5-*O*-GTs were differentially expressed as shown in Figs. 3 and 6 [1], then gomphrenin-I cannot be the most abundant betacyanin. However, gomphrenin-I has been claimed to be the most abundant betacyanin (Table S9) [1]. The following reasons make the claim unfeasible: 1) at least, in the case of *H. megalanthus*, there was no 6-*O*-GT expression observed by Xie et al. [17], 2) betanidin-5-*O*-glucosyltransferase (B5GT) and betanidin-6-*O*-glucosyltransferase (B6GT) share only 19% amino acid sequence identity suggesting that these enzymes are paraphyletic evolutionarily even if they are present in the same plant [20], and 3) betanin (betanidin-5-*O*-glucoside) has not been shown so far to convert into gomphrenin-I (betanidin-6-*O*-glucoside) via any enzymatic or non-enzymatic step. Further, the presence of amaranthin in pitaya has not been established so far through extensive spectroscopic characterisation and quantification [12–15] though Zhou et al. [1] claimed to have detected it. Amaranthin biosynthesis is completed only after glucuronylation at 2′-OH of betanin. Since Zhou et al. [1] did not report data on UDP-glucuronyltransferase and Xie et al. [17] also could not find any upregulated glucuronyltransferase gene, except for a down-regulated one in *H. megalanthus* peel, it is very unlikely that amaranthin was detected by Zhou et al. [1] in RR and GW samples, especially when other researchers had not detected it before in *Hylocereus* cacti [9, 12, 13].

### Anthocyanins and *ANS* profiling fall short of established standards

Betalains are tyrosine-derived metabolites, whereas anthocyanins are phenylalanine-derived. In plants, anthocyanidin synthase (ANS) converts colourless leucoanthocyanidins into anthocyanidin pigments [21]. Only after this step does glucosylation and formation of downstream compounds take place [21]. Consequently, it is generally believed that ANS is the most crucial point of separation between anthocyanin and betalain biosynthesis in plants, the other two crucial points being the convergence of one of the transcription factors involved in betalain biosynthesis, and deregulation of arogenate dehydrogenase to favour more tyrosine synthesis at the cost of phenylalanine [6]. Therefore, anthocyanins and betalains are widely accepted to be mutually exclusive, even though *ANS* is expressed in betalain-accumulating plants. In *Mirabilis jalapa*, a 69 amino acid truncated and catalytically inactive ANS is expressed, with the truncation involving a part of the active site [22]. Also, *ANS* is present intact but not expressed in betalain-accumulating *Spinacia oleracea* and *Phytolacca americana* [23]. Therefore, any finding contrary to the long-held and well-supported concept of mutual exclusivity of anthocyanins and betalains should be based on unquestionable evidence. Zhou et al. [1] did not include any *ANS* in Fig. 5 [1], but the expression of one *ANS* (cluster-864.105069) was presented in Fig. 6 [1]. However, the main concern here is that the lone *ANS* whose differential expression data is given in Fig. 6 [1] is not a functionally validated protein. Furthermore, its expression is not commensurate with anthocyanin content presented in Table S9 [1]. For example, RR peel has about ten times more total anthocyanins (all the differentially expressed anthocyanins taken together) than RR pulp (Table S9 in [1]), however, the *ANS* expression in both of them was not significantly different (Fig. 6 in [1]). Similarly, RR peel has about eight times more anthocyanins than GW peel (Table S9 in [1]), but the *ANS* expression was the same in both the samples (Fig. 6 in [1]). It may not be even required to look for downstream metabolite formation or corresponding gene expression, if *ANS* expression itself is implausible, because ANS acts as a catalyst that transforms colourless compounds/precursors into corresponding coloured products that exhibit characteristic spectra which are different from that of its precursors or flavonoids derived from the partially overlapping biosynthetic pathway [21, 24, 25]. Therefore, it is questionable as to how this lone *ANS* candidate whose expression does not correspond to the anthocyanin content can support the premise of anthocyanin accumulation in pitayas, let alone the co-occurrence of anthocyanins and betalains.

### Yellow colour formation appears to be not supported by the provided data

Zhou et al. [1] wrote on page 2, column 1, second paragraph that “….the color of the peel and pulp [of pitayas] which is contributed mainly by the pigment betalains and other secondary metabolites such as anthocyanins and carotenoids.” To support this statement, a reference was cited, although it does not report the characterisation of the pigment contents of vine cacti, but reports phenotypic and genomic characterisation. In the studied pitaya samples by Zhou et al. [1], YW peels were supposed to accumulate betaxanthins to ascribe their colour to, as contribution of anthocyanins (page 14, column 1, second paragraph, lines 11–13) and carotenoids (page 14, column 1, third paragraph, lines 7–9) in yellow colour formation was ruled out. However, there was no betaxanthin detected in the metabolite analysis data presented in Table S9 [1]. The presence of dopamine has been explained by Zhou et al. [1] as an indication of betaxanthin formation, however, the corresponding betaxanthin, miraxanthin V, which is yellow in colour, is not reported in Table 9 [1]. All the dopamine that has been reduced in yellow samples compared to green and red samples may not be completely attributed to betaxanthin formation, as was hypothesised by Zhou et al. [1]. On the other hand, betalamic acid is also greenish yellow in colour. However, it is also not listed in Table 9 [1] though others have reported its presence in *Hylocereus* spp. [9]. Any other amino acid, such as phenylalanine, can form a yellow betaxanthin (i.e. Phe-betaxanthin). However, such a yellow betaxanthin was also not identified in Table 9 [1]. Additionally, YW peels and pulps had betacyanins to be detected unlike betaxanthins which were simply assumed to be present but not detected by the same method of analysis that could detect betacyanins. In a separate study, Xie at al. [17], and Cejudo-Bastante et al. [18] reported 5–7 mg, and ca. 2.5 mg betaxanthins/100 g fresh peels, respectively, of *H. megalanthus* (or *Selenicereus megalanthus*) after colour breaking stage. So, taking into account all these inconsistencies, it is clear that the metabolite analysis method used by Zhou et al. [1] was not reliable enough to explain the colour formation in the studied samples.

## Data Availability

Not applicable.

## Abbreviations

5-O-GT: Betanidin-5-*O*-glucosyltransferase
6-O-GT: Betanidin-6-*O*-glucosyltransferase
ANS: Anthocyanidin synthase
B5GT: Betanidin-5-*O*-glucosyltransferase
B6GT: Betanidin-6-*O*-glucosyltransferase
BvDOD: *Beta vulgaris* DOPA-4,5-dioxygeanse
cD5GT: *c**yclo*-DOPA-5-*O*-glucosyltransferase
CYP76AD1: A cytochrome P450 protein with monooxygenase activity towards tyrosine and diphenol oxidase activity towards L-DOPA
DOD: DOPA-4,5-dioxygenase
GW: Green peel-white pulp
HPLC: High performance liquid chromatography
L-DOPA: L-3,4-dihydroxyphenylalanine
m/z: Mass to charge ratio
MS: Mass spectrometry
PgDOD: *Portulaca grandiflora* DOPA-4,5-dioxygenase
RR: Red peel-red pulp
UDP: Uridyl diphosphate
YW: Yellow peel-white pulp
